# Mechanical behavior of human breast tissues: *ex vivo* and *in silico* characterization

**DOI:** 10.1007/s10237-026-02045-x

**Published:** 2026-03-30

**Authors:** Ana Margarida Teixeira, António Diogo André, Marco Parente, Maria da Luz Barroso, Cristina Cunha, Horácio Costa, Pedro Martins

**Affiliations:** 1 Faculty of Engineering of University of Porto, Porto, Portugal; 2 Associated Laboratory of Energy, Transports and Aeronautics (LAETA), Institute of Science and Innovation in Mechanical and Industrial Engineering (INEGI), Porto, Portugal; 3 Gaia/Espinho Hospital Center: Plastic, Reconstructive, Craniomaxillofacial, Hand Surgery Service and Microsurgery Unit, Vila Nova de Gaia, Portugal; 4 Aragonese Foundation for Research and Development (ARAID), Instituto de Investigación en Ingeniería de Aragón (i3A), Universidad de Zaragoza, Zaragoza, Spain

**Keywords:** Breast tissues, Mechanical properties, Hyperelasticity, Indentation tests, Inverse finite element model

## Abstract

To study the mechanics of a biological tissue is crucial to understand its behavior under a variety of realistic loading conditions. In particular, an accurate estimation of the mechanical properties of breast tissues is essential to enhance current diagnostic techniques, such as mammography, and to improve clinical treatments, including tissue engineering approaches. This study focuses on the mechanical characterization of human breast tissues, harvested from women who underwent a mammary reduction surgery. *Ex vivo* experiments, consisted in indentation tests, were performed to determine the elastic properties. Additionally, using inverse finite element analysis, the hyperelastic properties were obtained for the Yeoh and Ogden (N = 3) constitutive models. The samples were grouped based on the characteristics of the female population (i.e., age, body mass index and menopausal status) and according to the breast side (tissue sample site). Significant differences in the Young’s modulus were only observed in association with age and menopausal status, in the first linear region (3.75–11.5% strain) of the experimental curve. In the second linear region (22.5–30% strain), although samples presented a higher stiffness, no significant differences were observed. Regarding the hyperelastic properties, Yeoh and Ogden (N = 3) models accurately fit the experimental data, presenting errors lower than 3.26% and 3.86%, respectively. In this work, the model developed successfully converged with a 3 mm of indentation (corresponding to 30% of deformation), enabling reliable analysis in the large deformations domain. The findings of this study provide valuable insights that can contribute to future clinical applications and research, including improvements in diagnostic techniques, treatments and esthetic reconstructions.

## Introduction

The breast is a vital organ for women, composed by different tissues including adipose, glandular and fibrous tissues as well as by the Cooper’s ligaments. All these components change along the life of a woman, due to several factors including age, menstrual cycle, pregnancy, menopause and lactation, among others. Considering the mechanics of breast tissues, Lorenzen et al. (2003) observed that the fibroglandular tissue was 2 times stiffer in the menstrual cycle. Moreover, when a pathology is present, the mechanical properties of the tissues change as well. It is known that a diseased tissue presents higher stiffness than normal tissue under compression (Teixeira and Martins 2023). Therefore, the mechanical behavior of breast tissues is relevant for clinical applications, with direct applications for breast lesions detection using techniques such as breast elastography (Teixeira and Martins 2023).

To better understand the mechanical behavior of breast tissues, finite element (FE) models have been developed as a tool to predict the *in vivo* behavior of the breast. However, those models depend highly on the mechanical properties defined for each tissue. Alongside with it, the patient-specific complex morphology of the breast and the difficulties of measuring the mechanical properties of the different types of tissues contribute to the challenge of modeling the breast for each patient (Ramião et al. 2016).

The mechanical properties of breast tissues have been presented in literature in terms of elastic and hyperelastic domains, assuming the breast tissues as isotropic and near incompressible (Wellman et al. 1999; Han et al. 2003; Samani et al. 2003; Delaine-Smith et al. 2016). If assuming also that the tissue is elastic, it can be characterized, under quasi-static compression, considering only the Young’s modulus. Under such assumptions, the mechanical behavior of breast tissues is independent of the geometry or boundary conditions and dependent only on the properties of the material (Krouskop et al. 1998; Griffin et al. 2016).

Regarding normal breast tissues (in the absence of a known disease), Krouskop et al. (1998) and Wellman et al. (1999) concluded that, under compression, adipose tissue had the lowest Young’s modulus. However, for Samani et al. (Samani et al. 2007, 2003), Matsumura et al. (2009) and (Umemoto et al. 2014), all normal tissues, i.e., adipose, glandular and fibrous tissues, had comparable stress–strain curves, with similar elastic modulus. Most studies compare normal with diseased tissues, being observed that pathologies are stiffer than normal tissues, with the malignant tumor being the stiffest (Krouskop et al. 1998; Wellman et al. 1999; Samani and Plewes 2007; Samani et al. 2007; Matsumura et al. 2009; Umemoto et al. 2014; Samani et al. 2003; Ramião et al. 2016).

A key consideration, regarding the measurement of the Young’s modulus, is the influence of the preconditioning. Krouskop et al. (1998) found that the stiffness of the tissues increases as the precompression increases. For instance, the tumor tissue was 5 times and 25 times stiffer than normal tissue, depending if the precompression level was set to 5% or 20%, respectively. This evidence confirms the nonlinear behavior of the tissues, which was also observed by Matsumura et al. (2009); Umemoto et al. (2014); Samani et al. (2003).

At large deformations, breast tissues exhibit viscoelastic behavior. Due to this property, loading and unloading curves are different as well as the curves from different cycles (Fung 1993), being this phenomenon known as hysteresis. Focusing on the hyperelastic properties of breast tissues, Samani and Plewes (2004) and Dempsey et al. (2021) successfully measured the hyperelastic parameters for adipose and fibroglandular tissues under indentation tests, using polynomial (N=2), Yeoh, Ogden (N=3) and Veronda–Westman models. Dempsey et al. (2021) concluded that there was no significant difference between the tissues, corroborating the use of a homogeneous model for large deformations. However, in these studies, the indentation depth was only 1 mm, which represented only 10% of deformation.

Through relaxation tests, Calvo-Gallego et al. (2020) measured the viscoelastic properties of adipose tissue from the breast and abdomen. They concluded that, under static loading, the mechanical behavior was similar, while under dynamic loading, it was different.

Another approach was used by Omidi et al. (2014), where the mechanical properties of human decellularized adipose tissue (DAT) and normal adipose breast tissue were measured. Performing indentation tests, they concluded that DAT from the breast had a similar stiffness (Young’s modulus of 3.460±1.210 kPa) when compared with adipose breast tissue (Young’s modulus of 3.250±0.910 kPa). In addition, the hyperelastic properties were obtained using inverse FE analysis with first-order polynomial, Yeoh, Ogden and Arruda–Boyce models, concluding that the Yeoh and Ogden models provide the best fit for breast DAT. The DAT was harvested from different regions of the body; however, they did not found significant differences between them and they inferred that DAT scaffolds are promising biomaterials for breast reconstruction, from a mechanical perspective.

Therefore, knowing the mechanical behavior of the tissues is essential to develop the most accurate biomaterial for tissue regeneration. Nevertheless, the comprehension of the mechanical properties of the tissues goes beyond that application. For the breast, the knowledge of its properties is essential to develop accurate FE models that are helpful to study the impact of bras (Sun et al. 2019; Zhang et al. 2022) on breast tissues mechanics, to evaluate the deformation of the tissues under physiological loading conditions (Haddad et al. 2016; Omidi et al. 2014), to estimate the postoperative shape of the breast after an implant insertion (Roose et al. 2005) or even to predict the exact location of a tumor (Babarenda Gamage et al. 2017).

In that regard, this study focuses on the characterization of human breast tissues, considering both the elastic and hyperelastic domains through *ex vivo* experimental tests (i.e., indentation tests) and *in silico* simulations. For the FE model, the Yeoh and Ogden (N=3) constitutive models were selected for their accuracy in modeling breast tissue behavior, amply documented in literature (Omidi et al. 2014; O’Hagan and Samani 2008, 2009). Notably, in this study, the displacement applied was higher than presented in literature (i.e., 30% vs. 10% deformation), which allows to better understand the mechanical response of breast tissues at larger deformations.

Furthermore, to understand the effect of the characteristics of the female population considered, samples were grouped according to age, body mass index (BMI) and menopausal status. Additionally, differences between the left and right breast were analyzed. In clinical practice, this information is relevant; however, in literature, the mechanical properties (typically) are not presented according to the population characteristics.

## Materials and methods

### Ex vivo experiments

#### Samples preparation

To evaluate the mechanical properties of breast tissue, human tissue samples were harvested from female patients, who performed mammary reduction surgery. All samples were collected with informed consent by the patients and in accordance with the ethical standards of Centro Hospitalar Vila Nova de Gaia/Espinho, with their approval for all the experimental protocols.

The samples were collected from both right and left breasts, from the lower outer quadrant. As soon as samples were harvested, they were immersed in saline solution and kept refrigerated ($\approx 10^{o}C$) until further analysis. The samples were collected with a minimum dimension of 50x50 $\hbox {mm}^2$, by, at least, 10 mm. After samples had been collected, the mechanical tests were performed as soon as possible, i.e., within the same day or in the day after, if the samples were harvested at late afternoon, with a maximum storage time of 12 h.

**Fig. 1:**
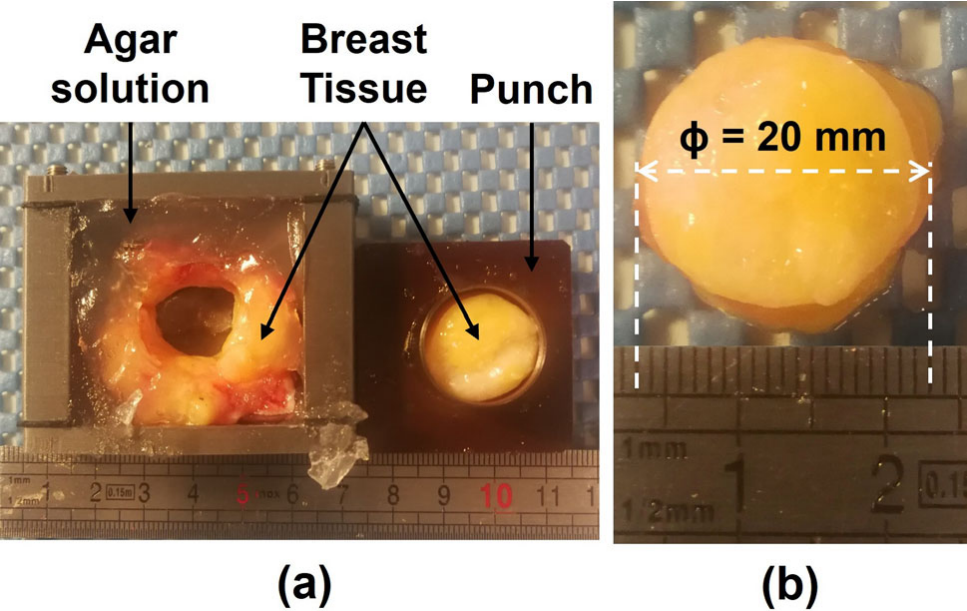
Specimens Preparation: **a** Tissue sample embedded in agar solution and the punch with the specimen inside, right after cutting it; **b** final tissue specimen with diameter of 20 mm

To prepare the samples for the experiment, an agar solution ($\approx$3.5%(w/w)) was used to immerse the original specimen and avoid movement (Samani et al. 2003). Sample confinement and the mechanical stabilization provided by the surrounding agar hydrogel, made it possible to obtain specimens with regular dimensions. A punch with 20 mm of diameter was used to cut each individual specimen, which had approximately a height of 10 mm. Figure 1 illustrates the methodology used. The dimensions of each sample were defined based on the study of Delaine-Smith et al. (2016), where the geometry (thickness and diameter) of agarose samples was investigated in relation with the diameter of the indenter. The authors had concluded that an adequate specimen geometry would present a ratio $\ge$4:1 between the diameter of the sample and the diameter of the indenter and a ratio $\ge$1:1 and $\le$2:1 between the thickness of the sample and the diameter of the indenter.

#### Mechanical characterization

**Fig. 2:**
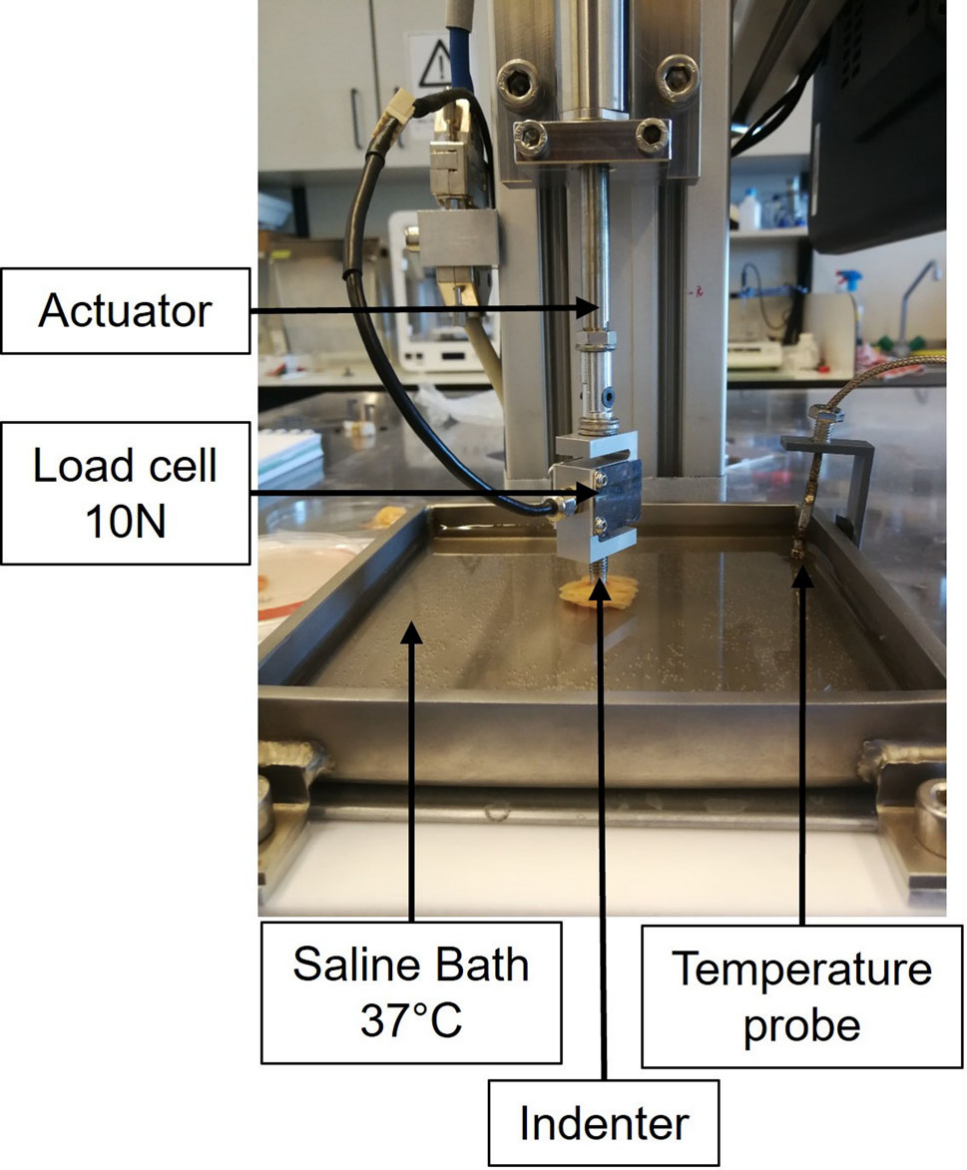
Mechanical setup used to perform the mechanical tests. The different components are represented, including the actuator, load cell, indenter/punch, the temperature probe and the saline bath

To carry out the mechanical tests, a custom-made machine composed by a load cell of 10 N and an actuator with a load capacity of 12 kg and a resolution of $3.05\times 10^{-4}$ mm were used. Indentation tests were performed using a flat-ended cylindrical indenter with a diameter of 5 mm. In addition, the samples were tested in a saline bath at $37^{o}C$, to mimic the physiological temperature. In figure 2, the setup scheme is presented.

From a previous study of the group (Teixeira and Martins 2020), it was concluded that preconditioning had an influence on the mechanical properties of agarose gels. Therefore, preconditioning was also applied to the breast tissue samples, minimizing the effect of hysteresis. A two-step mechanical protocol was defined as: (I) preconditioning: 20 cycles at 10% strain amplitude and 30% strain/min; (II) stress-relaxation test, in which each sample was loaded up to 30% strain at a rate of 30% strain/min, hold for 600 s at the final position and then unloaded up to 0% strain at the same rate.

#### Data analysis approach

The force–displacement data were digitally acquired at a sampling rate of 100 Hz. Due to hardware limitations, including low relative resolution of the force compared to displacement, interpolation was applied to regularize the data of individual samples. This method approximates discrete data by a continuous function, which overcomes the lack of points recorded, especially in the beginning of the test. In this way, the statistical treatment could be carried out properly considering the following:

1. it considers each experimental curve, $c^{j}_i$
  $$\begin{aligned} j \in \left\{ 1,...,m \right\} : j,m \in N \end{aligned}$$
  of the form
  $$\begin{aligned} c^{j}_{i}=(f_{i},d_{i}) \end{aligned}$$
  with a total of i
  $$\begin{aligned} i \in \left\{ 1,...,n \right\} : i,n \in N \end{aligned}$$
  experimental data points, as an individual experiment
2. it considers that all the curves $c^{j}$ describe the same phenomenon, i.e., the same testing protocol is applied to (*n*) identical samples

After regularization, it is possible to apply a point-wise statistical treatment to the curves, calculating the mean curve $c^{mean}$ representative of each group and statistical quantities such as the standard deviation (std).

Through the force–displacement (*f*, *d*) curve, the stress–strain $(\sigma ,\epsilon )$ curve can be obtained, given that all samples have the same geometry. The stress was calculated based on the indenter area, and the strain was obtained through the displacement of the indenter and the thickness of the sample.

From the stress–strain curve of each specimen, the Young’s modulus ($E_{mean}$) was calculated (Eq. 1) considering the slope of the first and second linear regions (from 3.75% to 11.5% and from 22.5% to 30%, respectively—defined by selecting from the mean curves the common portions found in the linear regions).

1 $$\begin{aligned} E=\frac{\sigma }{\epsilon } \end{aligned}$$

#### Statistical analysis

Samples were categorized based on patient-specific information. The group categories were: (i) *age* < 50 years or *age* > 50 years (older adults), (ii) *BMI* $< 25~\hbox {kg/m}^2$ or *BMI* $> 25~\hbox {kg/m}^2$ (overweight), (iii) Pre-M (premenopausal) or Post-M (postmenopausal), and (iv) breast of the left or right side.

For each group, the elastic properties were analyzed in the first and second linear regions of the mean curve, and significant differences were studied for each group at each linear region. Firstly, Shapiro–Wilk test was performed to investigate the normality of the data. Given that the assumption of normality was not met for most of the data and that there is more than one sample per patient in each group, the Wilcoxon signed-rank test was used to assess the occurrence of significant differences between the considered groups. Moreover, correlation between groups was assessed, applying the Spearman correlation test.

The p-values were adjusted for multiple testing using the Benjamini–Hochberg false discovery rate (FDR) method. Therefore, the p-values reported in this study refer to the adjusted p-values.

Statistical analyses were performed using SPSS (IBM), with a significance level of 0.05.

### Inverse FEM for hyperelastic parameters calibration

*In silico* simulations were based on the experimental profile of the indentation tests, focusing only on the loading ramp of the protocol. To develop the model, a flat and cylindrical indenter with 5 mm of diameter was modeled as a rigid body. The breast tissue sample, with 20 mm of diameter and 10 mm height, was defined using hexahedral mesh elements (C3D8H—8 node linear brick, hybrid formulation). One quarter of the tissue sample is composed by 21,200 elements and 23,709 nodes. It was defined as incompressible material, due to the high fluid content of the tissues, and as isotropic (Ramião et al. 2016), a common assumption in the literature for breast tissues.

**Fig. 3:**
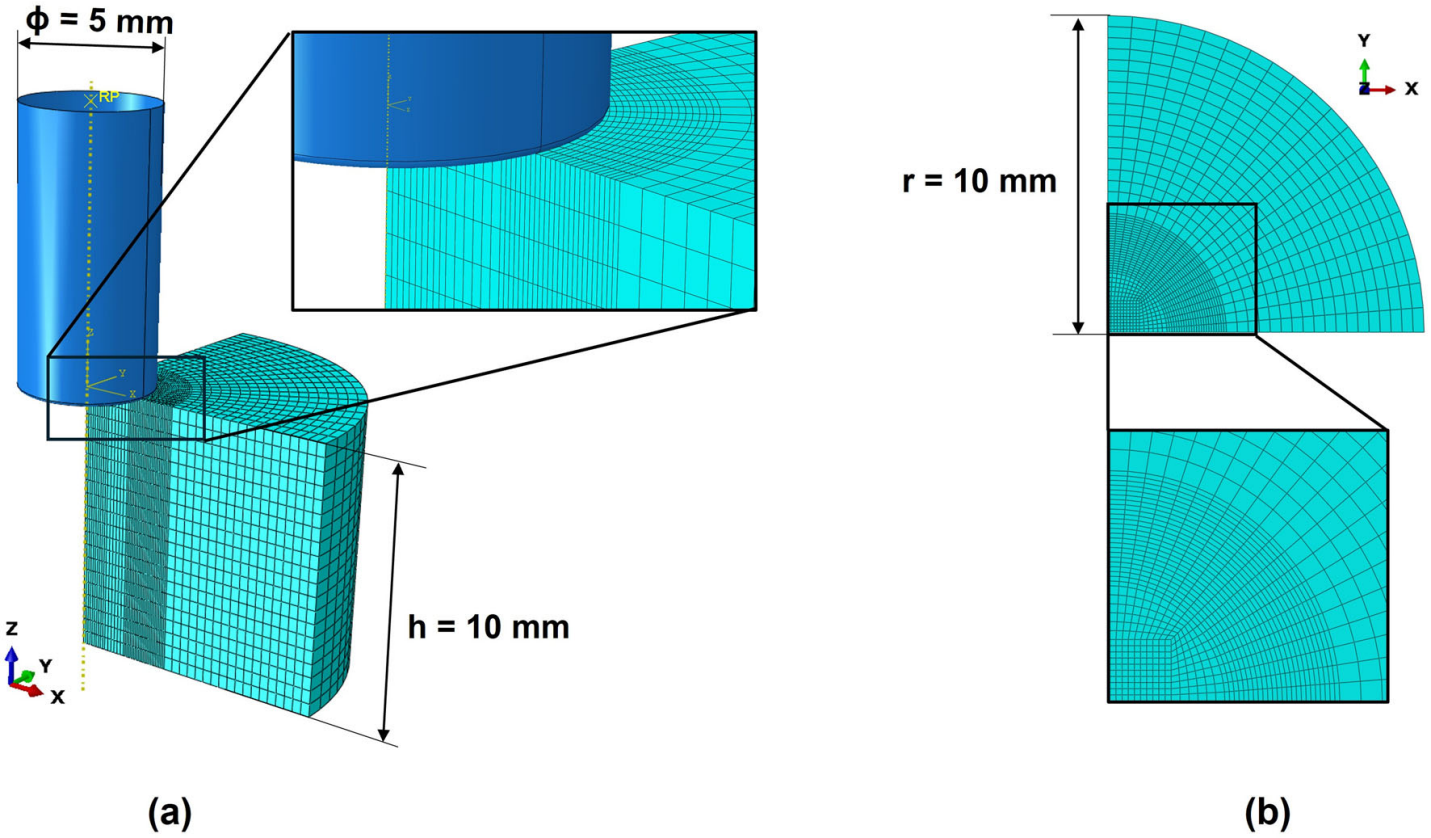
**a** View of the indenter-breast tissue assembly, with representation of one quarter of the tissue sample, composed by 21,200 linear hexahedral elements of type C3D8H. Magnification of the interaction region between the indenter and tissue sample. **b** top-view of one quarter of the tissue sample mesh, with a magnification of the region of interest (i.e., the region where the indenter touches the tissue)

In Fig. 3, the FE model is presented, including the mesh of the tissue sample and the rigid indenter. The mesh of the tissue sample was analyzed in terms of mesh sensitivity, using the Richardson’s extrapolation. The method is described in Appendix A, showing that the solutions produced by the different grids are within the asymptotic range of convergence. The mesh selected for the following steps of this study was the medium grid, showing a good compromise between the errors obtained and the computational time. Moreover, the mesh sensitivity analysis was performed using the Yeoh model and the properties obtained for the *BMI* $< 25~\hbox {kg/m}^2$ group, since it presents the highest Young’s modulus at large deformations (i.e., second linear region). Seeing that represents an extreme case, it is possible to state that the error of the mesh discretization will always be lower.

Using one quarter of the model, the boundary conditions in the sample included a displacement equal to zero in: (i) z-direction at the bottom; (ii) y-direction on x-plane; (iii) x-direction on y-plane. Moreover, on the indenter, only the displacement in z-direction was allowed. In addition, the contact between the indenter and the sample was defined as frictionless to mimic the experimental test.

**Fig. 4:**
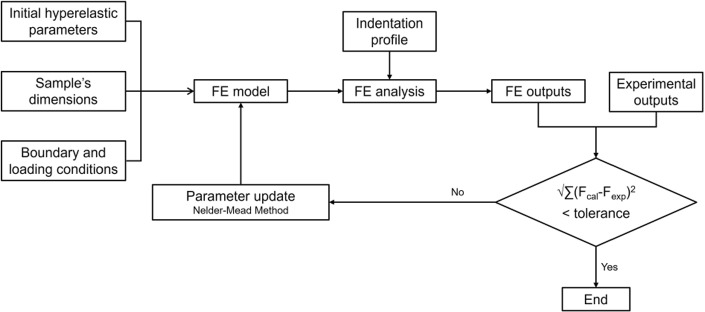
Workflow of the inverse FE method to obtain the hyperelastic material properties

Based on the developed FEM model, an inverse FE method was applied in order to obtain the hyperelastic properties of the breast tissues, under indentation. In Fig. 4, a workflow of the steps taken to the inverse FEM is presented. Firstly, the initial hyperelastic parameters (which were arbitrary), the dimensions of the sample and the boundary and loading conditions were defined into the FE model. Secondly, the indentation profile was defined and the FE analysis was performed. Then, the output of the simulation (($(F^{cal}_i$, $d_i$) curve) was compared with the output of the experimental tests, the ($F^{exp}_i$, $d_i$) curve. Through the root sum squared (RSS) error between the calculated force and the experimental force, for each displacement point, the difference between the simulated and experimental data can be calculated. If that difference is higher than the tolerance, defined by the user, then the hyperelastic parameters are updated. The updated FE model goes under analysis again, and the comparison with the experimental outputs is performed. Only when the difference between the simulated and experimental curve meets the tolerance ($RSS\le tol$), the iterative process is interrupted, achieving the final hyperelastic parameters.

To update the hyperelastic parameters, the Nelder–Mead optimization method was used to minimize the objective function (Eq. 2) defined as:

2 $$\begin{aligned} \min \sum _{i}^{} (\sigma ^{cal}_i-\sigma ^{exp}_i)^2 \end{aligned}$$

where $\sigma ^{exp}_i$ is the experimental stress and $\sigma ^{cal}_i$ is the calculated stress (from the model equation), in each point *i*.

The Nelder–Mead method is a commonly used optimization technique due to its ease of use and robustness, since it does not use derivatives and it can handle discontinuities (O’Hagan and Samani 2008). It is based in the simplex concept, in which the objective function is evaluated at the vertices of a simplex (i.e., a geometric shape with N+1 vertices in N dimensions). Through operations like reflection, expansion, contraction and shrinkage, the shape and position of the simplex are iteratively modified, until the diameter of the simplex is less than a specific tolerance, i.e., until convergence (Nelder and Mead 1965).

The hyperelastic parameters were obtained for the Yeoh and Ogden (N=3) models. The strain-energy function for the Yeoh material model is given by Eq. (3), while the strain-energy function for the Ogden material model is given by Eq. (4) (Martins et al. 2006).

3 $$\begin{aligned} \psi = \sum _{i=1}^{3} C_i(I_1-3)^i + p(J-1) \end{aligned}$$

where $C_i$ are the material hyperelastic parameters, $I_1 = \sum _{i=1}^{3}\lambda _i^2$, with $\lambda _i$ being the principal stretches, *p* is the hydrostatic pressure acting as a Lagrange multiplier and $J=det(F)$ with *F* being the gradient of deformation. For incompressible materials, J=1.

4 $$\begin{aligned} \psi = \sum _{i=1}^{N}\frac{\mu _i}{\alpha _i}(\lambda _1^{\alpha _i}+\lambda _2^{\alpha _i}+\lambda _3^{\alpha _i}-3) + p(J-1) \end{aligned}$$

where $N=3$, $\mu _i$ and $\alpha _i$ are the material hyperelastic parameters, $\lambda _1$, $\lambda _2$ and $\lambda _3$ are the principal stretches, *p* is the hydrostatic pressure acting as a Lagrange multiplier and $J=det(F)$ with *F* being the gradient of deformation. For incompressible materials, J=1.

For both material models, the parameters were left unconstrained during the optimization method (i.e., Nelder–Mead method) with the goal to achieve the best overall fitting to the experimental data. However, this might not guarantee the stability of the material, which needs to be evaluated afterward. For each set of coefficients, Drucker stability was tested by evaluating the material under uniaxial, biaxial and planar deformation modes, ensuring that the material behavior is physically realistic.

For an incompressible material, the Drucker stability assumes, as requirement, the following inequality: $\partial \sigma _{ij}\,\partial \varepsilon _{ij} > 0$, where $\partial \sigma _{ij}$ and $\partial \varepsilon _{ij}$ represent the change in stress and strain, respectively. Knowing that $\partial \sigma _{ij} = D_{ik}\partial \varepsilon _{kj}$, where $D_{ik}$ is the tangent material stiffness, the inequality becomes $D_{ik}\partial \varepsilon _{kj}\partial \varepsilon _{ij} > 0$, which is satisfied when $D_{ik}$ is positive definite. In terms of principal stresses and strains, the inequality can be written as $\partial \sigma _{1}\partial \varepsilon _{1}+\partial \sigma _{2}\partial \varepsilon _{2}+\partial \sigma _{3}\partial \varepsilon _{3} > 0$. Since the material is incompressible, the hydrostatic pressure can be any value, without affecting the strains. A convenient choice is $\sigma _{3}=\partial \sigma _{3}=0$, which simplifies the expression to $\partial \sigma _{1}\partial \varepsilon _{1}+\partial \sigma _{2}\partial \varepsilon _{2} > 0$. In the form of a matrix, it can be written as:

$$\begin{aligned} \begin{bmatrix} \partial \sigma _{1} \\ \partial \sigma _{2} \end{bmatrix} = \begin{bmatrix} D_{11} & D_{12} \\ D_{21} & D_{22} \end{bmatrix} \begin{bmatrix} \partial \varepsilon _{1} \\ \partial \varepsilon _{2} \end{bmatrix} \end{aligned}$$

Since $D_{ik}$ must be positive definite, it is necessary that the trace ${{\,\textrm{Tr}\,}}(D_{ik})$ and determinant $\det (D_{ik})$ are both positive, which are verified using Abaqus (Smith 2009). The check on the stability of the material is performed for the three deformation modes, for a nominal strain range of −0.9 to 30 at intervals of 0.01, being the matrix $D_{ik}$ computed at each increment. Therefore, the material is stable if the inequalities are met.

The FE model and FE analysis as well as the stability test were developed in Abaqus software (Dassault Systèmes, SE, Vélizy-Villacoublay, France) (Smith 2009)), while the workflow and the optimization algorithm were developed in Matlab (MathWorks, Natick, MA, USA, (Inc. 2022)).

## Results

A total of 33 specimens were obtained from 8 patients and were tested under indentation tests to assess the elastic and hyperelastic properties.

### Ex vivo experiments

**Fig. 5:**
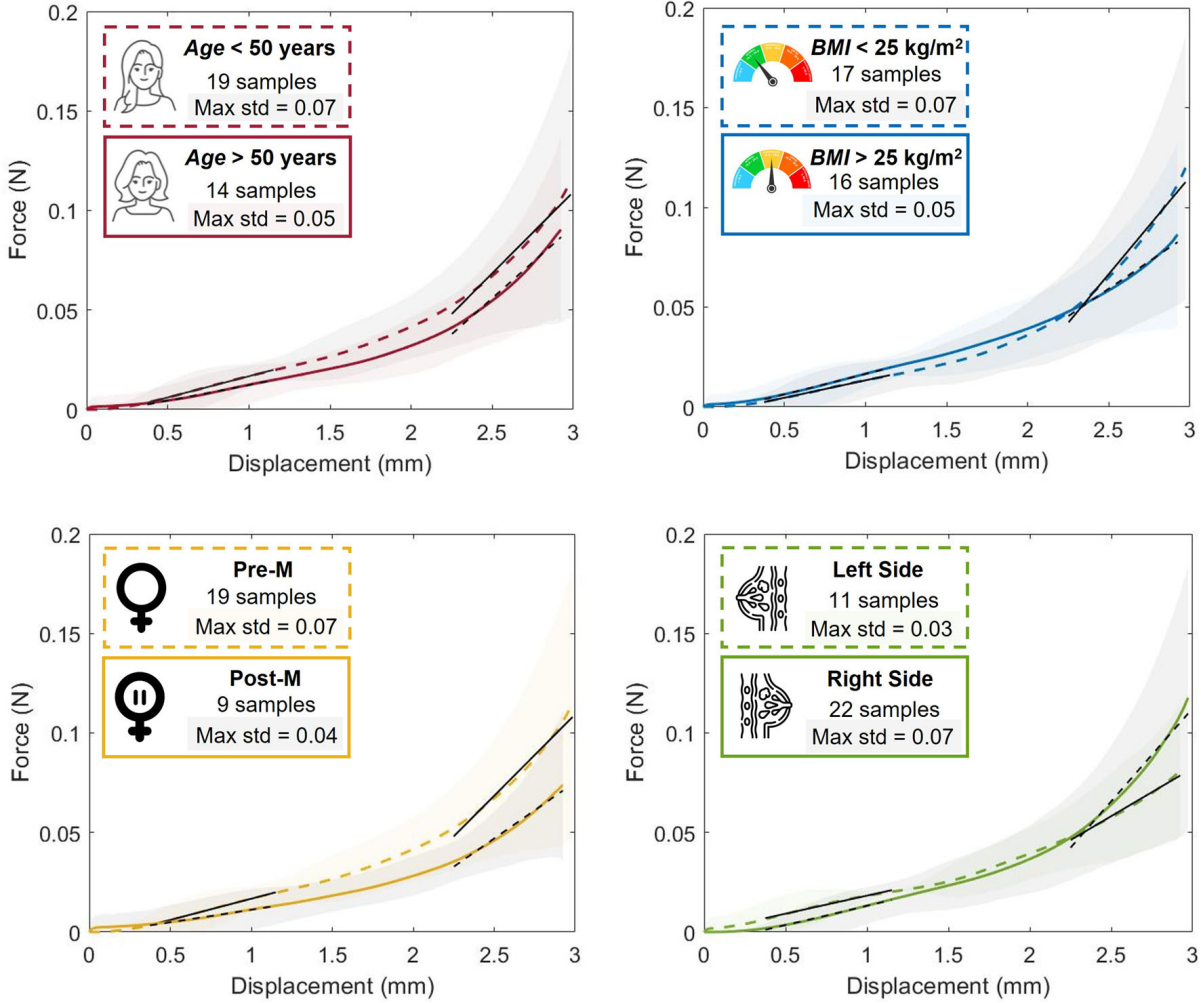
Mean force–displacement curves for each group analyzed. Top-left: Age group, i.e., *age* > 50 years (full line) and *age* < 50 years (dashed line); Top-right: BMI group, i.e., *BMI* $< 25~\hbox {kg/m}^2$ (full line) and *BMI* $> 25~\hbox {kg/m}^2$ (dashed line); Bottom-left: Menopausal status group, i.e., postmenopausal (full line) and premenopausal (dashed line); Bottom-right: Breast side group, i.e., right (full line) and left (dashed line). For each group, it is presented the number of samples as well as the maximum standard deviation, which corresponds at the highest displacement, in all curves. The shaded region around the mean curve represents the standard deviation area, and the black lines (full and dashed) represent the slope at each linear region

Samples were grouped according to the characteristics of the female population considered, such as age, BMI and menopausal status, as well as according to which breast the sample was harvested, i.e., right or left breast. For each group, the mean force–displacement curves were obtained (Fig. 5).

Through the experimental curves of each specimen, the Young’s modulus was calculated using Eq. (1) and considering two linear regions of the curve: from 3.75 to 11.5% of strain and from 22.5% to 30% of strain. For both linear regions, the mean Young’s modulus was obtained, being presented in Table 1 for each group.

**Table 1:** Young’s modulus results (mean±std) for the first and second linear regions of the force/stress vs. displacement/strain curve (n = number of samples)

		Young’s Modulus, ($\times 10^{-3}$) MPa	
		1^st^ linear region	2^nd^ linear region
*Age (years)*	> 50 (n = 14)	9.35±1.48*	42.17±7.18
	< 50 (n = 19)	13.07±1.08*	44.28±8.65
*BMI* ($\hbox {kg/m}^2$)	> 25 (n = 16)	12.07±1.29	32.67±7.09
	< 25 (n = 17)	10.95±1.35	53.47±8.44
Menopause	Post-M (n = 9)	6.88±1.80*	32.85±8.87
	Pre-M (n = 19)	13.07±1.08*	44.28±8.65
Side	Right (n = 22)	11.64±1.10	51.09±7.83
	Left (n = 11)	11.20±1.79	27.98±4.99

*Significantly different between the same group, with a p-value $\le$ 0.05

**Fig. 6:**
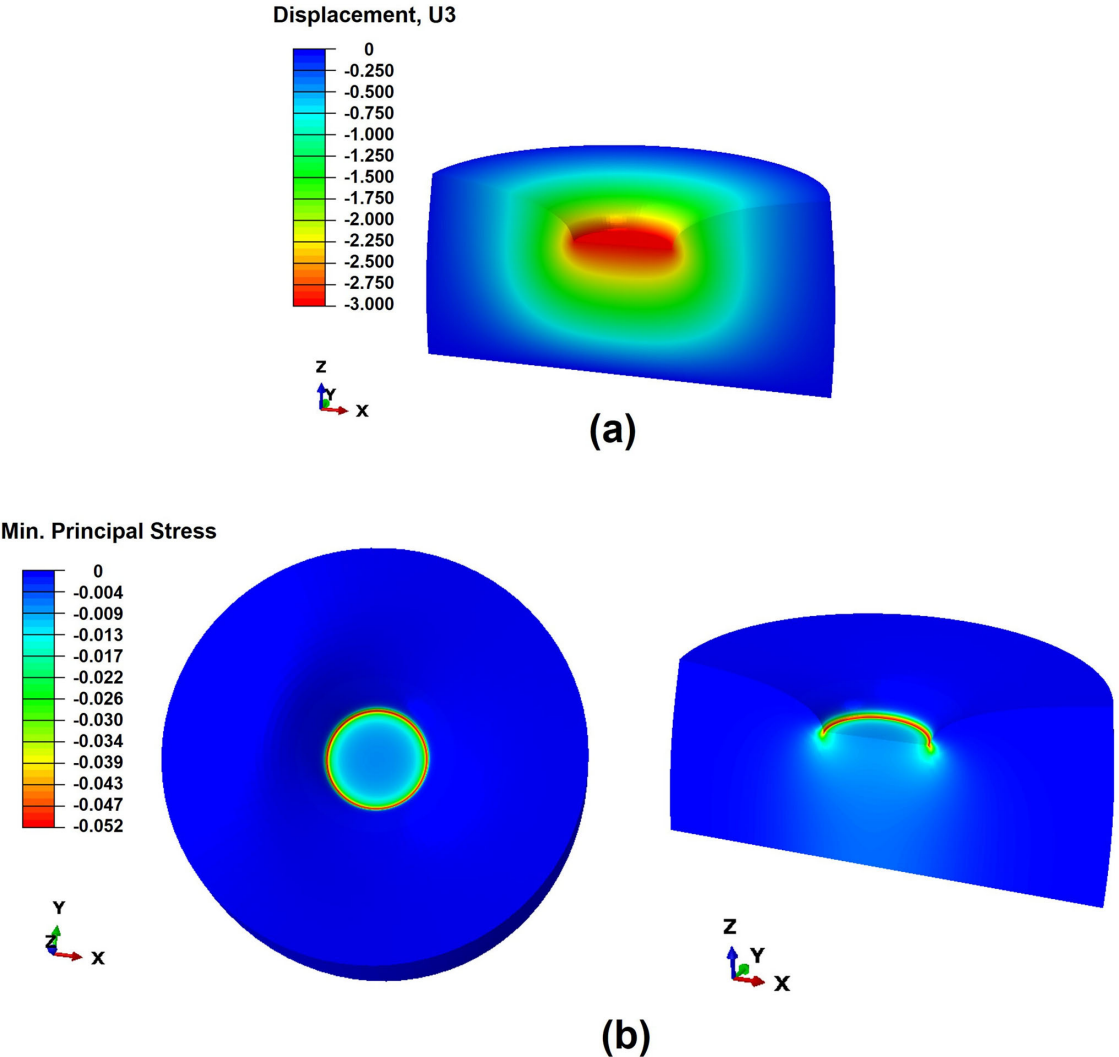
Results of a simulation: **a** displacement in z-axis (mm); **b** minimal principal stress (MPa) in the whole sample and in a cut view. Since indentation is applied in the $-z$ direction, the most negative value corresponds in fact to the highest stress, represented in red

The only significant differences found were in the age and menopausal status groups and in the first linear region, with a p-value $\le$ 0.05 for both groups. Women with age lower than 50 years present a stiffer breast tissue than older women as well as postmenopausal women when comparing with premenopausal women.

**Fig. 7:**
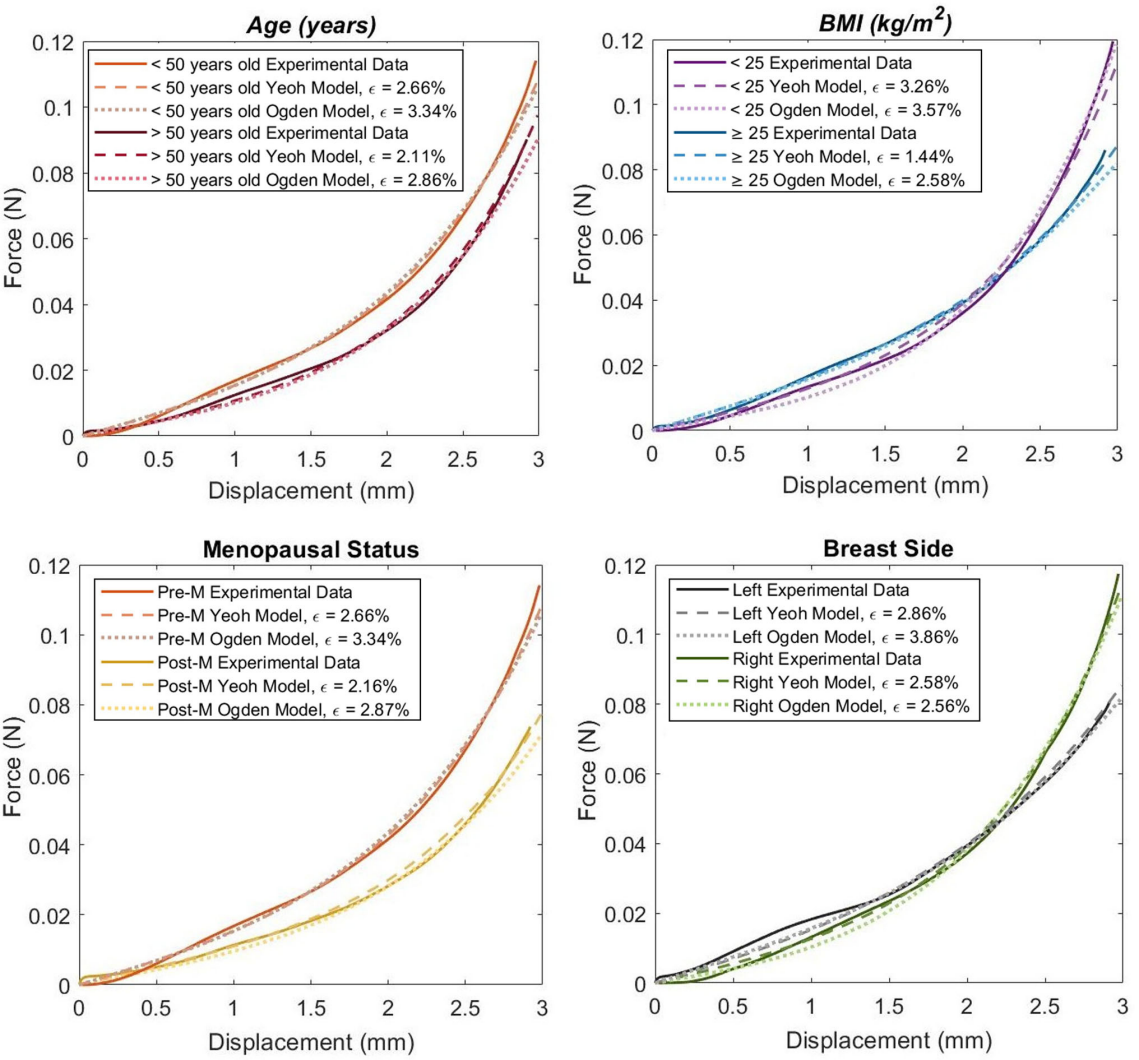
Experimental curves (full lines) and simulated curves for Yeoh (dashed lines) and Ogden (N = 3) (pointed lines) models. Top-left: Age group; Top-right: BMI group; Bottom-left: Menopausal status group; Bottom-right: Breast side group

When comparing the stiffness in the two linear regions, the second linear region presents higher values of Young’s modulus; however, no significant differences were found in any group.

Regarding the correlation between groups, taking into account the results from the Spearman correlation test, the groups are only correlated in the first linear region. A BMI higher or equal than 25 $\hbox {kg/m}^2$ showed to be correlated with the groups of premenopausal women and with women under 50 years old, with a p-value=0.004. In reality, the groups of premenopausal women and with age lower than 50 years are composed exactly by the same samples, evidencing that no woman under 50 years old was in menopause. Moreover, the group of postmenopausal women and the group of samples harvested from the left breast were correlated with a p-value=0.016.

However, after adjustment of the p-values, no statistically significant correlations between the groups were observed.

In appendix B, the complete correlation tables are presented as well as the adjusted p-values, respectively, for the first linear region (Table 4) and the second linear region (Table 5).

### Inverse FEM for hyperelastic parameters calibration

Besides the elastic properties obtained directly from experimental results, calculating the slope of the experimental curves, the hyperelastic properties were also assessed for each group.

A FE model was developed to mimic the experimental protocol, specifically the loading curve. A displacement of 3 mm was defined, which corresponded to 30% strain, and the hyperelastic coefficients were obtained for the Yeoh and Ogden (N=3) models.

In Fig. 6, a deformed sample is presented, including the outputs of the displacement imposed as well as the minimal principal stress.

Using inverse FE method, the hyperelastic properties were firstly defined based on literature (Dempsey et al. 2021), and through an iterative process, they were updated until the difference between the experimental and simulated curves was within the desired tolerance.

The final curves are presented in Fig. 7, for each group, including the experimental curve and the hyperelastic curves for the Yeoh and Ogden (N=3) models.

Both hyperelastic models provide a good fitting to the experimental curves, with errors between 1.44% and 3.26% for the Yeoh model and between 2.56% and 3.86% for the Ogden (N=3) model.

In Tables 2 and 3, the final coefficients for the Yeoh and Ogden (N=3) models are presented, respectively.

**Table 2:** Hyperelastic parameters, in $N/mm^2$, for the Yeoh model

		Yeoh Coefficients		
		$C_{1}\times 10^{-6}$	$C_{2}\times 10^{-6}$	$C_{3}\times 10^{-6}$
*Age (years)*	> 50	799.14	1726.42	5143.47
	< 50	1200.00	2000.00	4000.00
*BMI* ($\hbox {kg/m}^2$)	> 25	1333.33	1137.78	2568.89
	< 25	890.00	2200.00	5800.00
Menopause	Post-M	889.00	900.00	3900.00
	Pre-M	1200.00	2000.00	4000.00
Side	Right	938.19	2168.35	5701.17
	Left	1200.00	2222.22	829.44

**Table 3:** Ogden model (N = 3) coefficients, being $\mu _i$ in $N/mm^2$ and $\alpha _1$ dimensionless

		Ogden Coefficients (N=3)					
		$\mu _1\times 10^{-6}$	$\alpha _1$	$\mu _2\times 10^{-6}$	$\alpha _2$	$\mu _3\times 10^{-6}$	$\alpha _3$
*Age (years)*	> 50	−21537.81	2.15	18911.25	4.98	4307.56	−4.85
	< 50	−21598.50	2.21	20042.47	4.26	4003.58	−5.26
*BMI* ($\hbox {kg/m}^2$)	> 25	−20720.62	2.07	19459.53	4.09	3988.57	−3.87
	< 25	−21827.24	2.13	19455.05	5.40	4025.95	−6.11
Menopause	Post−M	−23527.78	2.04	21027.78	3.97	4083.33	−4.39
	Pre−M	−21598.50	2.21	20042.47	4.26	4003.58	−5.26
Side	Right	−26326.21	2.92	21570.90	5.62	6165.84	−4.40
	Left	−20720.37	2.07	19459.72	4.08	3988.52	−3.87

## Discussion

The mechanical properties of a tissue are crucial to deeply understand and ultimately model its physical behavior, although they change under different circumstances. For example, in the presence of a pathology, the mechanical properties are affected significantly, typically increasing the stiffness when compared with a healthy breast tissue.

Enhancing the knowledge about the mechanical behavior of breast tissues in any scenario is essential for a variety of clinical applications. It not only aids in improving diagnosis techniques but also contributes to the development of more accurate treatments and esthetic procedures, including tissue engineering approaches.

Previous studies have characterized breast tissues using *in vivo*, *ex vivo* and *in silico* experiments. Besides the biological variability across individuals, mechanical properties are also influenced by the testing protocols. For example, it has been shown that preconditioning in *ex vivo* experiments influences the stiffness of a tissue (Krouskop et al. 1998; Umemoto et al. 2014).

In this work, human breast tissue samples were harvested from 8 patients that performed a mammary reduction surgery. A total of 33 samples were tested and categorized according to age, BMI, menopausal status and the breast side. The *ex vivo* experiments revealed that the elastic moduli were higher in the second linear region, when comparing to the first linear region, regardless of the group considered. However, only the age and menopausal status groups show significant differences between the subgroups in the first linear region.

As expected, older women presented a breast tissue less stiff than younger women, likely due to aging-related changes in breast composition, with adipose tissue becoming predominant (Shim et al. 2023; Lin et al. 2023). As adipose tissue is the softest tissue in the breast, the stiffness decreases.

Similarly, postmenopausal women have a softer breast tissue than premenopausal women. This is also attributed to the breast being primarily composed of adipose tissue, which is intrinsically related to the aging process. In menopause, women suffer hormonal changes, including a decrease of estrogen (Lin et al. 2023), which leads to a decrease of the glandular tissue (Nedel et al. 2024), as well as a decrease in the collagen content of the connective tissue (Calleja-Agius and Brincat 2012). Therefore, the overall breast density decreases, being the prominent tissue, adipose tissue (Shim et al. 2023; Lin et al. 2023). Age and menopausal status are thus intrinsically related in women, which was also reflected in the Spearman correlation analysis. Between the groups age < 50 years and premenopausal, the correlation coefficient was 1.000, which indicates that the groups are indistinguishable (i.e., composed by the same specimens). Nevertheless, increasing the number of samples, these groups could become distinct if a patient under 50 years old had premature menopause or, on the other hand, if a patient above 50 years old had delayed menopause. Therefore, the number of samples might be a limitation of this study. In addition, through the Spearman correlation test, the groups of women under 50 years old and with BMI higher or equal to 25 are correlated, showing that most of the younger women, and hence premenopausal women, in this study were overweight.

Afterward, through Benjamini–Hochberg FDR method, the p-values were adjusted and no statistically significant correlations between the groups were detected, suggesting that the previous findings (i.e., based on unadjusted p-values) might represent false-positive results. However, it is important to notice that a more reliable analysis could be achieved with a larger sample size.

Elastic properties were not significantly influenced by BMI and breast laterality. Nevertheless, at larger deformations, the samples from patients with a BMI lower than $25~\hbox {kg/m}^2$ and from the right side exhibited the highest stiffness. At lower deformations, the stiffer tissue was observed in younger and premenopausal women.

Even though in literature, the mechanical properties of breast tissues are not typically grouped according to the patient-specific of the female population considered, the results of this study are in accordance with the quantitative values reported. Depending on the testing definitions, the Young’s modulus of adipose tissue varied from 18±7 kPa to 24±6 kPa for Krouskop et al. (Krouskop et al. 1998), from 4.8±2.5 kPa to 17.4±8.4 kPa for Wellman et al. (Wellman et al. 1999), from 0.69±0.19 kPa to 19.08±4.99 kPa for Umemoto et al. (Umemoto et al. 2014) and from 0.7±0.2 kPa to 17.3±4.8 kPa to Matsumura et al. (Matsumura et al. 2009), while Samani et al. obtained values of 1.9 kPa (Samani et al. 2003) and 3.25±0.91 kPa (Samani et al. 2007). For glandular tissues, some studies reported a similar stiffness to the adipose tissue (Samani et al. 2003, 2007; Umemoto et al. 2014; Matsumura et al. 2009), whereas others, such as Krouskop et al. (Krouskop et al. 1998) and Wellman et al. (Wellman et al. 1999), found out that the glandular tissue was significantly stiffer, with a Young’s modulus ranging from 17.5±8.6 kPa to 271.8±167.7 kPa.

The samples in the present study were obtained from mammary reduction surgeries, indicating that they were composed by adipose and glandular tissues, which was also confirmed by histology analysis using hematoxylin and eosin (H&E) staining. However, further histological analysis was not possible, given the small number of slices per sample, which constitutes a limitation of this study. Nevertheless, Dempsey et al. (Dempsey et al. 2021) reported that assuming mixed tissues as homogeneous is a valid assumption, as no significant differences in mechanical properties were found between mixed tissue and pure tissue types, such as adipose and fibroglandular. Consequently, for the estimation of the hyperelastic parameters, samples were assumed as homogeneous, isotropic and incompressible.

To have a better understanding of the mechanical behavior of the breast tissue, the hyperelastic properties were obtained using the Yeoh and Ogden (N=3) models, as they were previously shown to provide the best agreement with experimental data of breast tissue (Omidi et al. 2014; O’Hagan and Samani 2008, 2009). In this work, when comparing the experimental and simulated curves, errors between 1.44% and 3.26% for the Yeoh model and between 2.56% and 3.86% for the Ogden (N=3) model were obtained, indicating a good fit for both models.

The hyperelastic coefficients obtained in this study are in accordance with those reported by Dempsey et al. (Dempsey et al. 2021), being in the same order of magnitude. However, Dempsey et al. (Dempsey et al. 2021) only applied 1 mm of displacement to the FE model, while in the present study a deeper displacement was applied to accurately replicate the experimental protocol, thus enabling the investigation of the mechanical behavior under larger deformations.

Using the Drucker stability test, all the coefficients obtained were found stable for uniaxial, biaxial and pure shear tensions, showing that the material properties are stable and represent a realistic physical behavior, for a wide range of strains.

The main limitation of the hyperelastic characterization using the inverse FE scheme considered is the sensitivity of the optimization algorithm (Nelder–Mead) to the initial seeds chosen for the hyperelastic coefficients’ estimation. These seed values were selected taking the existing literature (Dempsey et al. 2021) as a starting point. In this study, the viscoelastic behavior of breast tissues is not presented due to setup limitations leading to unreliable data acquisition, both in quantity and quality. The low relative resolution, mentioned before in Sect. 2, contributed for the few data obtained during the relaxation time. This constitutes an additional limitation of this study, which will be addressed in a future work, by increasing the relaxation time during the experimental test.

Nevertheless, the outputs of this study, the elastic and hyperelastic properties of breast tissues, have numerous applications. Using FE models, real-world scenarios can be simulated *in silico* to investigate the behavior of the breast. For example, the impact of supporting bras in walking, jogging or even after a surgery can be analyzed. In terms of diagnostic techniques, such as mammography, the influence of the different testing parameters can be simulated and critically evaluated, aiming for an increased reliability and accuracy of the medical diagnosis. Another example is the evaluation of implants that can be added to the breast model enabling the possibility of accessing their biomechanical impact in a multitude of scenarios. In regenerative medicine, the implantation of scaffolds can also be simulated, making it possible to predict the mechanical behavior of both the breast and the implanted structure, which is crucial for its success.

One strength of this work is that, by having the mechanical properties grouped by women’s characteristics, allows the development of specific solutions that can be tailored based on the patient-specific characteristics.

## Conclusion

The mechanical properties of breast tissues are essential to clinical application, not only for improving diagnostic techniques’ accuracy, but also for treatments and reconstructions, including tissue engineering approaches.

In this study, the elastic and hyperelastic behavior of harvested human breast samples was characterized by *ex vivo* experiments and *in silico* simulations. Samples were grouped according to the characteristics of the female population considered, allowing the evaluation of their influence on tissue mechanics. Age and menopausal status were found to significantly affect the stiffness of the breast tissues, whereas the BMI and the breast side did not show a significant influence on the stiffness.

Moreover, this study expands the current state of the art on the mechanical behavior of breast tissues, to the domain of very large deformations. The range of deformations explored, using the proposed inverse FE method ($>30\%$) was significantly larger when compared with the available literature ($\approx 10\%$), matching the actual experimental protocol followed. This empowers researchers to address a wider range of simulations with many real-life applications.

The main limitation of this work is related to the number of samples, which was constrained by the limited number of patients that underwent surgery, during the time of this research.Ideally, it would be set a higher number of samples, from more patients, in order for all the groups to have a more representative sampling. Besides the categories used in this study, it would be interesting to extend the analysis to other breast quadrants (i.e., besides the outer lower quadrant), to assess the mechanical properties of each region of the breast.

Nevertheless, the findings reported in this study provide valuable insights for a wide range of future clinical and research applications.

## Figures and Tables

**Fig. 8:**
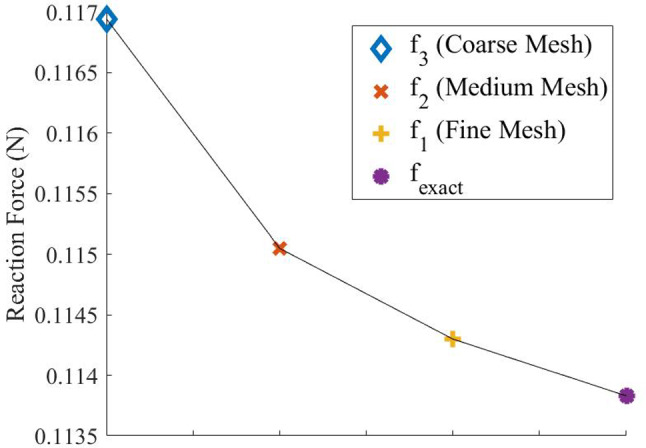
Reaction force (N) for each grid spacing, with a refinement ratio of 3. As the grid spacing reduces, the reaction force gets close to the exact value, which corresponds to a zero grid spacing

## Data Availability

No datasets were generated or analyzed during the current study.

## Appendix A: Mesh sensitivity: Richardson extrapolation

A grid convergence study was carried out using Richardson’s Extrapolation. For that, three levels of refinement were investigated, using a constant refinement ratio (r) equal to 3.

Based on Roache (1994); Roy (2010), firstly, the order of grid convergence was calculated using the following equation:

A1 $$\begin{aligned} p=\ln (\frac{f_{3}-f_{2}}{f_{2}-f_{1}})/ln(r) \end{aligned}$$

where $f_{i}$ are the discrete solutions of three different grids ($i=1,2,3$) with uniform spacings: $f_{3}$ is the coarse grid solution, $f_{2}$ is the medium grid solution and $f_{1}$ is the fine grid solution.

**Table 4:** Spearman correlation results based in the Young’s modulus of the first linear region

		Age > 50	Age < 50	Side Left	Side Right	BMI $\ge$ 25	BMI < 25	Pre-M	Post-M
Age > 50	Corr. Coefficient	1.000	−0.024	−0.173	−0.204	−0.095	0.116	−0.024	−0.100
	Sig. (2-tailed)		0.935	0.612	0.483	0.748	0.692	0.935	0.798
	N	14	14	11	14	14	14	14	9
	Adjusted p-value		0.935	0.826	0.819	0.861	0.859	0.935	0.826
Age < 50	Corr. Coefficient		1.000	−0.464	0.418	0.671	−0.164	1.000	0.517
	Sig. (2-tailed)			0.151	0.075	0.004^**^	0.529		0.154
	N		19	11	19	16	17	19	9
	Adjusted p-value			0.415	0.405	0.054	0.819		0.415
Side Left	Corr. Coefficient			1.000	−0.200	−0.345	0.273	−0.464	−0.767
	Sig.(2-tailed)				0.555	0.298	0.417	0.151	0.016^*^
	N			11	11	11	11	11	9
	Adjusted p-value				0.819	0.671	0.819	0.405	0.861
Side Right	Corr. Coefficient				1.000	0.409	−0.150	0.418	0.117
	Sig. (2-tailed)					0.116	0.567	0.075	0.765
	N				22	16	17	19	9
	Adjusted p-value					0.002	0.002	0.002	0.002
BMI $\ge$ 25	Corr. Coefficient					1.000	−0.362	0.671	−0.150
	Sig. (2-tailed)						0.169	0.004^**^	0.700
	N					16	16	16	9
	Adjusted p-value						0.415	0.054	0.859
BMI < 25	Corr. Coefficient						1.000	−0.164	0.217
	Sig. (2-tailed)							0.529	0.576
	N						17	17	9
	Adjusted p-value							0.819	0.819
Pre-M	Corr. Coefficient							1.000	0.517
	Sig. (2-tailed)								0.154
	N							19	9
	Adjusted p-value								0.415
Post-M	Corr. Coefficient								1.000
	Sig. (2-tailed)								
	N								9
	Adjusted p-value								

*Significantly different with a p-value $\le$ 0.05; **significantly different with a p-value $\le$ 0.01. In addition, adjusted p-values are also presented, obtained through Benjamini–Hochberg FDR method. Age is reported in years and BMI in $\hbox {kg/m}^2$

The exact solution (i.e., at zero grid spacing) was then estimated using the two finest grids, as follows:

A2 $$\begin{aligned} f_{exact}=f_{1}+\frac{f_{1}-f_{2}}{r^{p}-1} \end{aligned}$$

One metric, to check the asymptotic range, is the grid convergence index (GCI), which can be defined as:

A3 $$\begin{aligned} GCI=\frac{F_{s}\left| \varepsilon \right| }{r^{p}-1} \end{aligned}$$

where $F_s$ is the factor of safety, which is equal to 1.25 for comparisons over three or more grids, and $\left| \varepsilon \right|$ is the relative error. The relative error between the fine (1) and medium (2) grids is calculated as:

A4 $$\begin{aligned} \varepsilon =\frac{f_{1}-f_{2}}{f_{1}} \end{aligned}$$

while between the medium (2) and coarse (3) grids is calculated as:

A5 $$\begin{aligned} \varepsilon =\frac{f_{2}-f_{3}}{f_{2}} \end{aligned}$$

Following the methodology above and comparing the reaction force of each grid, we could conclude that the solutions are within the asymptotic range of convergence.

In figure 8, the plot of each reaction force of each grid as well as the exact solution is presented.

The order of convergence obtained for these results was 0.853, and the exact solution was 0.114 N.

The grid convergence index, in percentage, for grids 1 and 2 was 0.524% ($GCI_{12}$), while for grids 2 and 3 was 1.329% ($GCI_{23}$).

Using both values, we can check if the solutions are in the asymptotic range of convergence. The ratio $GCI_{23}/(r^p GCI_{12})$ was 0.994, which is approximately one, indicating that the solutions are within the asymptotic range of convergence.

## Appendix B: Correlation tables

See Tables 4 and 5.

**Table 5:** Spearman correlation results based in the Young’s modulus of the second linear region

		Age > 50	Age < 50	Side Left	Side Right	BMI $\ge$ 25	BMI < 25	Pre-M	Post-M
Age > 50	Corr. Coefficient	1.000	−0.002	0.045	−0.116	−0.332	−0.178	−0.002	0.533
	Sig. (2-tailed)		0.994	0.894	0.692	0.246	0.543	0.994	0.139
	N	14	14	11	14	14	14	14	9
	Adjusted p-value		0.994	0.994	0.994	0.830	0.994	0.994	0.830
Age < 50	Corr. Coefficient		1.000	−0.264	0.074	−0.376	−0.034	1.000	−0.483
	Sig. (2-tailed)			0.433	0.764	0.151	0.896		0.187
	N		19	11	19	16	17	19	9
	Adjusted p-value			0.994	0.994	0.830	0.994		0.830
Side Left	Corr. Coefficient			1.000	−0.564	0.027	0.091	−0.264	0.133
	Sig. (2-tailed)				0.071	0.937	0.790	0.433	0.732
	N			11	11	11	11	11	9
	Adjusted p-value				0.830	0.994	0.994	0.994	0.994
Side Right	Corr. Coefficient				1.000	0.312	−0.069	0.074	−0.283
	Sig. (2-tailed)					0.240	0.794	0.764	0.460
	N				22	16	17	19	9
	Adjusted p-value					0.830	0.994	0.994	0.994
BMI $\ge$ 25	Corr. Coefficient					1.000	−0.074	−0.376	0.050
	Sig. (2-tailed)						0.787	0.151	0.898
	N					16	16	16	9
	Adjusted p-value						0.994	0.830	0.994
BMI < 25	Corr. Coefficient						1.000	−0.034	−0.217
	Sig. (2-tailed)							0.896	0.576
	N						17	17	9
	Adjusted p-value							0.994	0.994
Pre-M	Corr. Coefficient							1.000	−0.483
	Sig. (2-tailed)								0.187
	N							19	9
	Adjusted p-value								0.830
Post-M	Corr. Coefficient								1.000
	Sig. (2-tailed)								
	N								9
	Adjusted p-value								

*Significantly different with a p-value $\le$ 0.05; **significantly different with a p-value $\le$ 0.01. In addition, adjusted p-values are also presented, obtained through Benjamini–Hochberg FDR method. Age is reported in years and BMI in $\hbox {kg/m}^2$
